# Fucoxanthin ameliorates *Propionibacterium acnes*-induced ear inflammation in mice by modulating the IκBα/NF-κB signaling pathway and inhibiting NF-κB nuclear translocation

**DOI:** 10.1371/journal.pone.0322950

**Published:** 2025-05-07

**Authors:** Shan Jiang, Xiaoyan Peng, Yan Chen, Bingqi Dong, Hu Mao

**Affiliations:** Department of Dermatology, Renmin Hospital of Wuhan University, Wuhan City, Hubei Province, China; Cairo University, EGYPT

## Abstract

**Background:**

Acne vulgaris, a chronic inflammatory skin disorder, represents a pivotal research area in dermatology. Although fucoxanthin, a marine-derived carotenoid, displays potent anti-inflammatory activity, its therapeutic potential in acne pathogenesis remains underexplored.

**Objective:**

This study investigates fucoxanthin’s effects on *Propionibacterium acnes* (*P.acnes*)-induced auricular inflammation in mice, focusing on its modulation of the IκBα/NF-κB signaling axis and inhibition of NF-κB nuclear translocation.

**Methods:**

Inflammation in the ear of mice was induced using a *P.acnes* injection model. The anti-inflammatory effects of fucoxanthin were verified by evaluating the levels of erythema, pathological damage, and inflammatory factors in the mice ear. An in vitro model was constructed to explore the regulatory mechanism of IkappaBalpha (IκBα)/nuclear factor-kappaB (NF-κB) pathway by fucoxanthin.

**Results:**

Fucoxanthin alleviated *P. acnes*-induced inflammatory pathology, reducing ear erythema. Mechanistically, it preserved IκBα stability, suppressed NF-κB nuclear translocation, and decreased proinflammatory cytokine production.

**Conclusion:**

Fucoxanthin exerts anti-acne effects through coordinated inhibition of IκBα degradation and NF-κB nuclear translocation, establishing its potential as a targeted therapeutic agent for inflammatory acne.

## 1. Introduction

Acne is one of the most prevalent inflammatory skin conditions, frequently triggered by bacterial infections, with *P.acnes* assuming a pivotal role in its onset [1]. Primarily affecting adolescents, acne can persist into adulthood, characterized by manifestations like pimples, papules, pustules, and scarring [2]. The pathogenesis of acne unfolds through diverse factors within the follicular sebaceous gland unit, involving heightened sebum production, altered follicular keratinization, and colonization by *P.acnes*—a key player in the ensuing inflammatory cascade marked by the release of pro-inflammatory cytokines [3,4].

The progression of acne can culminate in chronic inflammation, hyperpigmentation, and scarring, substantially impacting patients’ well-being. Although antibiotics and retinoids have been widely used in the treatment of acne, the increasing resistance of *P. acnes* has become a significant concern. Prolonged use of tetracyclines has led to a rise in resistance, reducing their efficacy and limiting the use of antibiotics. Additionally, retinoids are associated with adverse drug reactions, such as skin dryness, erythema, and peeling caused by topical adapalene, as well as systemic side effects, including hepatotoxicity, elevated blood lipids, and teratogenicity from oral isotretinoin. These factors collectively increase the risk of treatment failure [5,6]. Hence, the quest for innovative pharmaceutical interventions is imperative in the realm of acne therapeutics.

**Fig 1:**
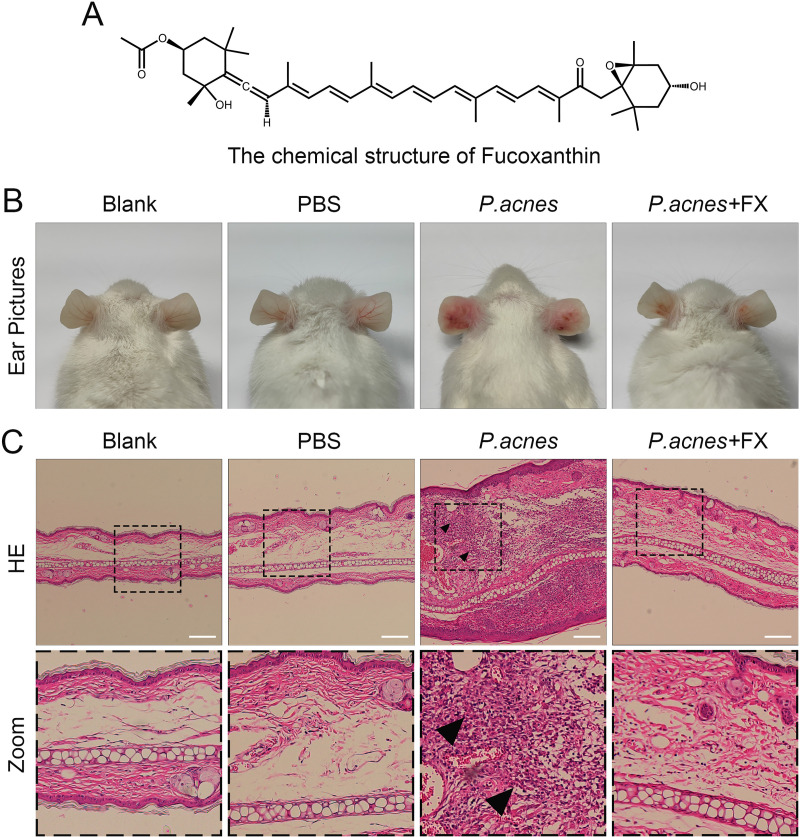
Fucoxanthin’s chemical structure and its effects on improving mice ear damage. (A) The chemical structural formula fucoxanthin; (B) Macroscopic photographs of mice ear; (C) HE staining of mice ear tissue (magnification 100X, scale bar = 100 μm. The black arrows indicate inflammatory cell infiltration and granuloma formation).

Fucoxanthin (FX, Fig 1A), a carotenoid found in marine plants, particularly abundant in brown algae and some microalgae, constitutes roughly 10% of natural carotenoid production [7,8]. Fucoxanthin is distinguished by specific allyl bonds and 5,6-mono epoxides, endowing it with robust anti-inflammatory, antioxidant, anti-apoptotic, anti-diabetic, and anti-tumor properties [9–11]. In terms of anti-inflammatory effects, fucoxanthin exerts protective roles in inflammatory diseases such as airway inflammation, sepsis, and non-alcoholic fatty liver disease through multiple mechanisms [12–14]. Notably, fucoxanthin has demonstrated efficacy in shielding the skin from UV-induced photoaging in mice models and enhancing skin vitality and anti-aging properties upon supplementation [15,16]. Given its potent cytoprotective attributes, the therapeutic promise of fucoxanthin in addressing acne warrants significant attention.

Nuclear factor-kappaB (NF-κB), a pivotal transcription factor, orchestrates a spectrum of physiological and pathological processes encompassing inflammation, cell proliferation, apoptosis, and immune responses [17,18]. In its inactive state, NF-κB forms a complex with IkappaBalpha (IκBα), hindering its nuclear translocation. Upon cellular stimulation, IκBα degradation liberates NF-κB, enabling it to enter the nucleus and modulate the transcription of genes linked to inflammation [19–21]. Research indicates that fucoxanthin, a compound derived from seaweed, can manifest anti-inflammatory effects in conditions like acute lung injury, enteritis, and vascular atherosclerosis by modulating the IκBα/NF-κB signaling pathway [22–24].

In this study, by establishing in vivo and in vitro models, it was hypothesized that fucoxanthin has a favorable anti-inflammatory effect on acne, and its underlying molecular mechanism may be related to the regulation of the IκBα/NF-κB signaling pathway, and that fucoxanthin may become a new therapeutic agent for acne.

## 2. Materials and methods

### 2.1. Preparation of bacteria

*P.acnes* (BeNa Culture Collection, Henan; CHN) was cultured at 37°C under anaerobic conditions (10% H_2_, 5% CO_2_, and 85% N_2_). The bacteria culture medium was supplemented with Dried Meat ParticleBroken Meat Medium Base (TOPBIO, Shandong, CHN).

### 2.2. In vivo infection model of *P.acnes* in mice

Male ICR mice (8 weeks, 34-40g) used for the experiment were obtained from the Laboratory Animal Center of the First Clinical College of Wuhan University and were housed in a SPF environment, and all mice had free access to water and food. All experiments were approved by our Laboratory Animal Committee and all procedures were performed in accordance with the guidelines for the care and use of laboratory animals published by the National Institutes of Health. *P.acnes* was collected by centrifugation and resuspended in PBS, and 20 μL of *P.acnes* bacteriophage (1 × 10^8^ Colony Forming Unit) was injected in each ear of the mice. The control group mice were injected with 20 μL of PBS in each ear. Three days before *P.acnes* injection, mice were pretreated with intraperitoneal fossilized fucoxanthin (HPLC ≥98%, MedChemExpress, New Jersey, USA, 100 mg/kg once a day). 24 hours after the injection of *P.acnes*, mice were anesthetized by intraperitoneal injection of sodium pentobarbital, and after photographs of the ears were taken, the mice were over-anesthetized in order to execute the mice and collect tissue samples. Ear swelling was assessed by measuring the thickness of the mice’s ears.

### 2.3. Hematoxylin and Eosin (HE) staining

Mice ear tissues were subjected to HE staining according to a standard protocol; briefly, the tissues were formaldehyde-fixed and then paraffin-embedded. The embedded samples were cut into 4 μm thick sections. Staining was performed using Hematoxylin and Eosin Staining Kit (Beyotime Biotechnology, Shanghai, CHN) according to the manufacturer’s instructions, and imaging was performed after staining was completed.

### 2.4. Inflammatory factor assay

Mice ear tissues weighing 0.1 g were immersed in 900 μL of physiological saline, and then the mixture was ultrasonically ground and centrifuged at 3000 g for 15 min to obtain tissue homogenates. For cell samples, cells were collected and centrifuged after sonication to collect the supernatant. A commercial ELISA kit (TNF-α, IL-1β, Abcam, Cambridge, UK) was used. Inflammatory factor levels were determined by measuring absorbance at 450 nm.

### 2.5. Cell culture and lipopolysaccharides (LPS) treatment

Human immortalized keratin-forming cells (HaCaT) were cultured in a DMEM medium containing 10% fetal bovine serum. The incubator conditions were 37°C and 5% CO_2_. Cells were cultured to a fusion level of 90% for modeling. Cells were stimulated using LPS (1 μg/mL, treated for 6 h) to mimic the inflammatory environment. For fumonisin treatment, cells were pretreated with 5 μM fumonisin for 24 h, followed by LPS treatment.

### 2.6. Cell viability assay

HaCaT cell viability was determined using the Cell Counting Kit-8 (Beyotime Biotechnology, Shanghai, CHN). Briefly, HaCaT cells were inoculated into 96-well plates. After 24h of incubation, the cells were incubated with different concentrations of fucoxanthin (0.01, 0.1, 0.5, 5, 25, 50, and 100 μM) for 24 h. Then LPS was added to the cells for 6 h. After completion of the treatment, the cells were incubated with CCK-8 reagent for 60 min at 37°C. The optical density values were detected at 450 nm to determine cell viability.

### 2.7. Western blot analysis

Western blot analysis was performed according to standard protocols. Total cellular proteins were extracted and nuclear and cytoplasmic proteins were extracted according to the instructions of Nuclear and Cytoplasmic Protein Extraction Kit (Beyotime Biotechnology, Shanghai, CHN). Cytoplasmic proteins were extracted. The extracted proteins were separated by electrophoresis on a 10% SDS-PAGE gel and then transferred to a PVDF membrane. The PVDF membrane was closed with 5% skimmed milk powder and then incubated with primary antibodies (Bcl-2, Bax, NLRP3, IκBα, p-NF-κB p65, GAPDH, H3; the antibodies were all diluted at a ratio of 1:1000 and purchased from Cell Signaling Technology, Massachusetts, USA) overnight at 4°C in a refrigerator. The next day PVDF membranes and corresponding secondary antibodies were incubated at room temperature for 1h, followed by protein band imaging with ECL luminescent solution.

### 2.8. Immunofluorescence

After HaCaT cells were processed according to the experimental protocol, they were fixed using formaldehyde for 15 min at room temperature, followed by closure with 10% goat serum at room temperature for 1 h. p-NF-κB p65 antibody (Abcam, Cambridge, UK) was added, incubated overnight, and washed the next day to add fluorescent secondary antibody and DAPI, and imaged under the fluorescence microscope.

### 2.9. Statistical analysis

All data are expressed as mean ± standard deviation (SD). The software involved in analyzing the data was GraphPad Prism 8 software (GraphPad Software, California, USA) and Image J. Statistical differences were compared using the Student’s t-test and one-way ANOVA, and differences of *P* < 0.05 were considered statistically significant.

## 3. Results

### 3.1. Fucoxanthin significantly ameliorated erythema and swelling in the ears of mice

The mice ear acne model was established through initial intradermal injection of *P.acnes* into bilateral auricular tissue. Photos of the mice ears taken 24 hours post-*P.acnes* injection revealed conspicuous red patches and swelling, mirroring clinical acne symptoms. Importantly, injecting PBS alone did not elicit similar responses. Following intraperitoneal administration of fucoxanthin as pretreatment, a marked reduction in erythema and edema was observed in murine auricular tissues compared to the *P.acnes*-challenged group (Fig 1B; SFig 1in S2 File, *P* < 0.001). Histopathological analysis demonstrated that *P.acnes* induced inflammatory cell accumulation and granuloma formation in the mice ears, phenomena reversed by fucoxanthin treatment (Fig 1C). These findings underscore the therapeutic promise of fucoxanthin in addressing mice ear acne.

### 3.2. Fucoxanthin reduces *P.acnes*-induced inflammation and cell apoptosis in mice ears

**Fig 2:**
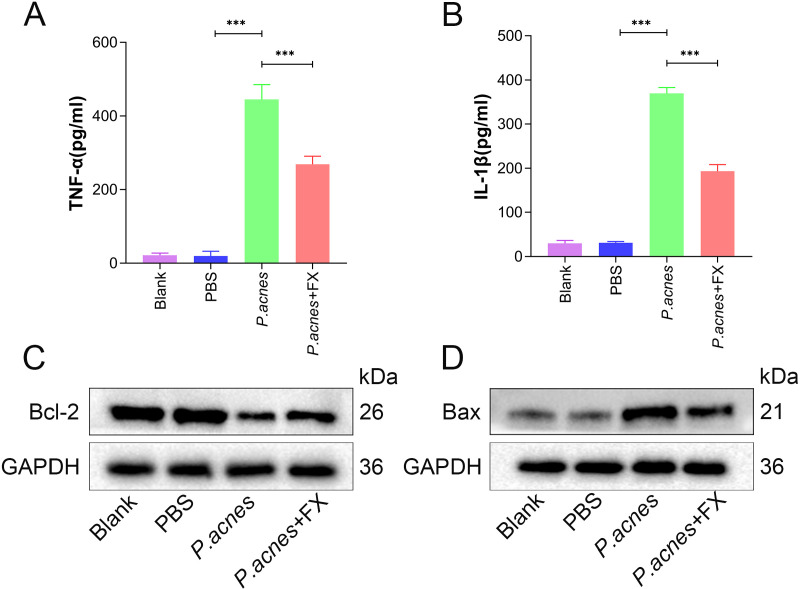
The impact of fucoxanthin on inflammation and apoptosis in mice ear. (A) Levels of TNF-α in mice ear tissue; (B) Levels of IL-1β in mice ear tissue; (C) Expression levels of Bax protein in mice ear tissue; (D) Expression levels of Bcl-2 protein in mice ear tissue. Values are expressed as mean ± SD. *** P < 0.001.

Inflammation and cell apoptosis are pivotal features of acne, prompting us to further assess changes in inflammation-related factors and apoptosis-related proteins in the ears of mice. Compared with the control group, the data showed a significant increase in the levels of the inflammatory factors TNF-α and IL-1β in the mice ear tissues 24 hours post-*P.acnes* injection, whereas fucoxanthin treatment decreased the levels of TNF-α and IL-1β (Fig 2A, B, P < 0.001). Furthermore, concerning cell apoptosis, the data showed that *P.acnes* promoted cell apoptosis, evidenced by a decrease in the anti-apoptotic protein Bcl-2 levels and an increase in the pro-apoptotic protein Bax levels. However, the alterations in Bcl-2 and Bax protein levels were partially reversed by fucoxanthin (Fig 2C, D; SFig 2A, B in S2 File, *P* < 0.01). These data indicate that fucoxanthin possesses inhibitory effects on *P.acnes*-induced inflammation and cell apoptosis.

### 3.3. Fucoxanthin enhances HaCaT cell viability and inhibits LPS-induced inflammation

**Fig 3:**
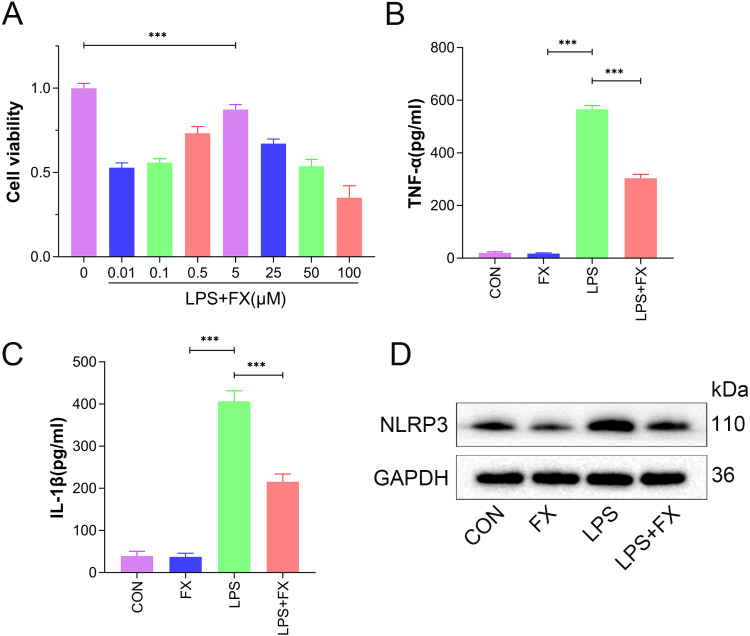
The inhibitory effect of fucoxanthin on inflammation in HaCaT cells. (A) Viability levels of HaCaT cells; (B) Levels of TNF-α in HaCaT cells; (C) Levels of IL-1β in HaCaT cells; (D) Expression levels of NLRP3 protein in HaCaT cells. Values are expressed as mean ± SD. *** *P* < 0.001.

To delve into the molecular mechanisms underlying the anti-inflammatory effects of fucoxanthin, the researcher established an in vitro model by stimulating HaCaT cells with LPS. Under LPS stimulation, the data showed that fucoxanthin significantly boosted the viability of HaCaT cells (Fig 3A, *P* < 0.001). Notably, at a concentration of 5 μM, fucoxanthin exhibited the highest cell viability in HaCaT cells. Consequently, subsequent cell experiments were conducted with a fucoxanthin concentration of 5 μM. By measuring the levels of TNF-α and IL-1β, the data showed that LPS induced a severe inflammatory response in HaCaT cells. Similarly, fucoxanthin reduced the levels of TNF-α and IL-1β in HaCaT cells, thereby inhibiting cellular inflammation (Fig 3B, C, *P* < 0.001). NLRP3, an inflammasome, serves as a crucial factor in sensing and mediating inflammation in the body. Following LPS stimulation, the data showed a significant increase in NLRP3 protein expression levels in HaCaT cells, which were subsequently inhibited by fucoxanthin (Fig 3D; SFig 3 in S2 File, *P* < 0.001).

### 3.4. Fucoxanthin inhibits the activation of the IκBα/NF-κB signaling pathway and suppresses NF-κB nuclear translocation

**Fig 4:**
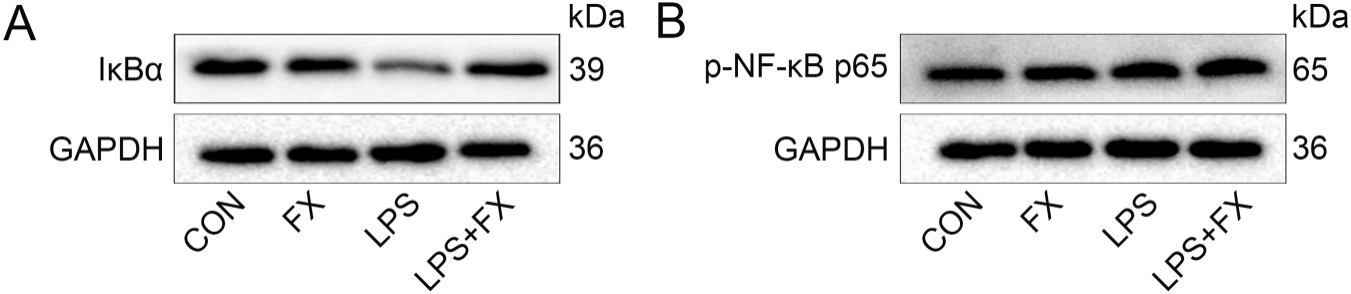
The regulatory effect of fucoxanthin on the IκBα/NF-κB signaling pathway. (A) Expression levels of IκBα protein in HaCaT cells; (B) Expression levels of p-NF-κB p65 protein in HaCaT cells.

**Fig 5:**
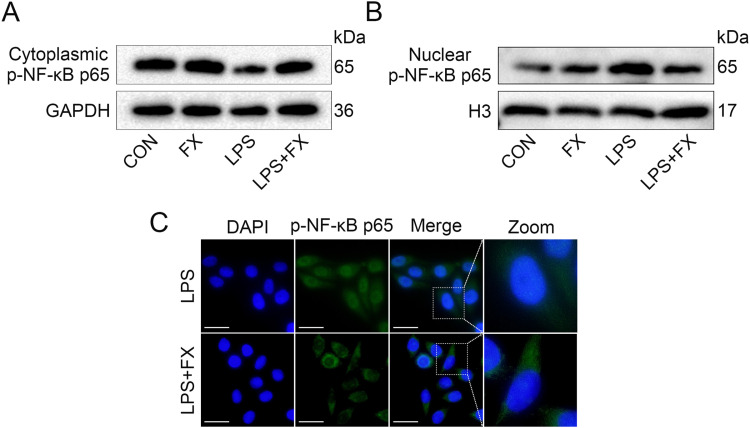
The inhibition of NF-κB nuclear translocation by fucoxanthin. (A) Expression levels of cytoplasmic p-NF-κB p65 protein in HaCaT cells; (B) Expression levels of nuclear p-NF-κB p65 protein in HaCaT cells; (C) Immunofluorescence detection of nuclear p-NF-κB p65 protein expression in HaCaT cells (magnification 400X, scale bar = 20 μm).

It is well known that the IκBα/NF-κB signaling pathway is closely linked to inflammatory responses, and studies have shown that fucoxanthin can exert anti-inflammatory effects in various inflammation-related diseases by modulating the IκBα/NF-κB signaling pathway. Therefore, the researcher investigated the effects of fucoxanthin on the IκBα/NF-κB signaling pathway in an acne model. Data indicate that under LPS stimulation, the protein expression levels of IκBα in HaCaT cells significantly decreased, while fucoxanthin was able to counteract the effects of LPS and restore the protein expression of IκBα (Fig 4A; SFig 4A in S2 File, *P* < 0.001). In an inflammatory environment, NF-κB dissociates from IκBα and translocates into the cell nucleus to activate downstream inflammatory factors. The data showed that both LPS and fucoxanthin had no significant impact on the overall protein expression levels of p-NF-κB p65 in HaCaT cells (Fig 4B; SFig 4B in S2 File). By separately extracting cytoplasmic and nuclear proteins, the data showed that after LPS stimulation, the protein expression of p-NF-κB p65 decreased in the cytoplasm and significantly increased in the nucleus. Compared to the LPS group, fucoxanthin promoted the expression of p-NF-κB p65 in the cytoplasm and inhibited its expression in the nucleus (Fig 5A, B; SFig 5A, B in S2 File, *P* < 0.001). Immunofluorescence experiments for p-NF-κB p65 protein indicated that fucoxanthin treatment reduced the expression levels of p-NF-κB p65 in the cell nucleus (Fig 5C). These data suggest that fucoxanthin inhibits the dissociation of the IκBα/NF-κB complex and suppresses the nuclear translocation of NF-κB.

### 3.5. Fucoxanthin inhibits NF-κB nuclear translocation in vivo

**Fig 6:**
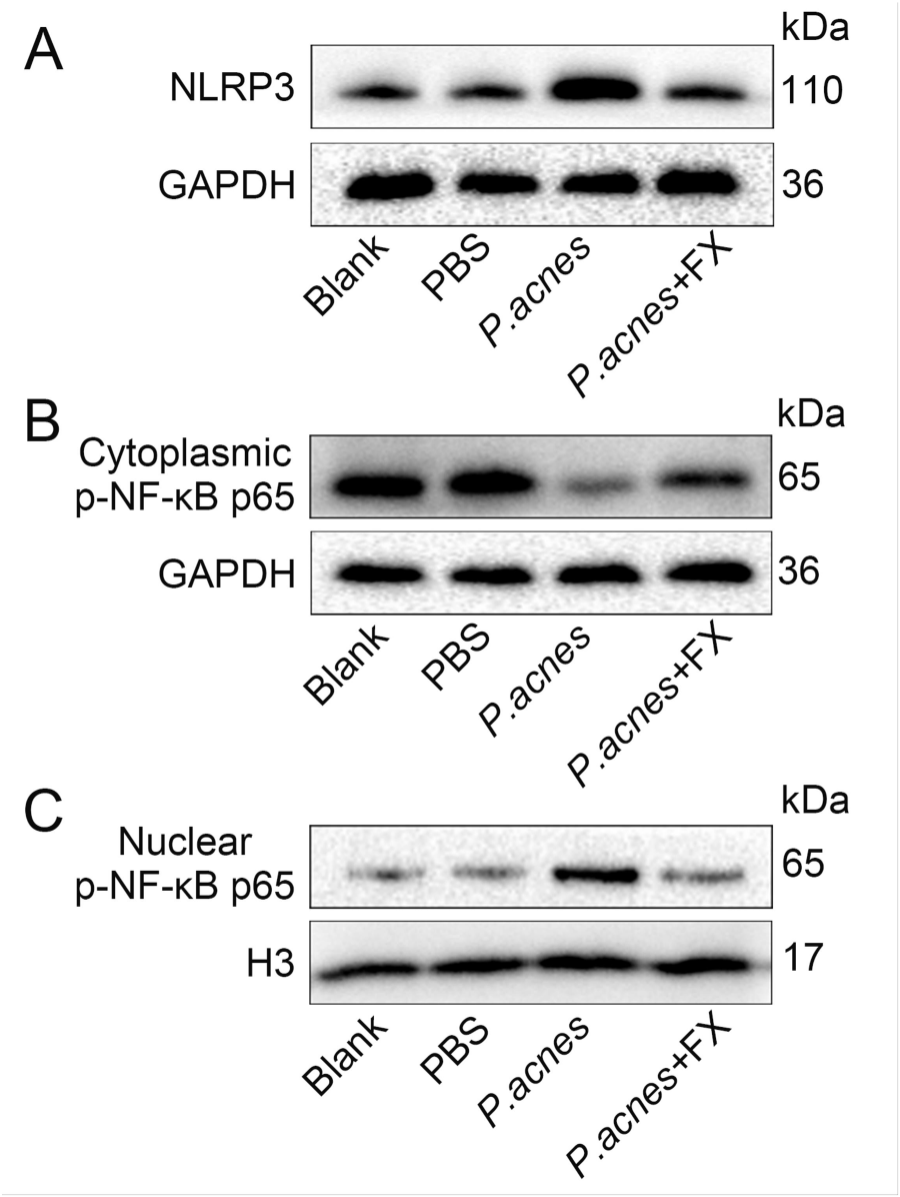
The regulatory effects of fucoxanthin on NLRP3 and NF-κB in mice ear tissue. (A) Expression levels of NLRP3 protein in mice ear tissue; (B) Expression levels of cytoplasmic p-NF-κB p65 protein in mice ear tissue; (C) Expression levels of nuclear p-NF-κB p65 protein in mice ear tissue.

Similarly, the researcher validated the regulatory effect of fucoxanthin on NF-κB in an in vivo model. Injection of *P.acnes* in the ears of mice promoted the expression of the inflammasome NLRP3 protein, while fucoxanthin significantly reduced the protein expression levels of NLRP3 (Fig 6A, SFig 6A in S2 File, *P* < 0.01). By extracting protein from the mice ear tissues, the data showed that *P.acnes* led to a decrease in the protein expression levels of p-NF-κB p65 in the cytoplasm and an increase in the nucleus, whereas treatment with fucoxanthin reversed the changes in p-NF-κB p65 protein (Fig 6B, C; SFig 6B, C in S2 File, *P* < 0.001). Therefore, fucoxanthin avoids the activation of downstream inflammatory factors and alleviates ear inflammation in mice by inhibiting the nuclear translocation of NF-κB.

## 4. Discussion

In this study, the researcher focused on exploring the mechanism of action of fucoxanthin in mice ear inflammation induced by *P.acnes*, with a particular emphasis on investigating its effects on improving inflammation by regulating the IκBα/NF-κB signaling pathway and inhibiting NF-κB nuclear translocation. The NF-κB signaling pathway plays a crucial role in the inflammatory process by regulating the release of inflammatory mediators, and fucoxanthin, as a natural compound, is believed to have potential anti-inflammatory effects. The research results reveal the key role of fucoxanthin in inflammation regulation, providing a new theoretical basis for further research on its clinical applications.

During the experiments, the researcher observed that fucoxanthin significantly alleviated the inflammatory lesions in mice ears induced by *P.acnes*, including reducing local swelling and erythema of inflammation, while also lowering the levels of inflammatory mediators. Compared to previous studies, this research further elucidates the mechanisms of fucoxanthin in inflammation regulation. Although earlier studies have indicated the anti-inflammatory potential of fucoxanthin [25], The study not only delved into its role in the NF-κB signaling pathway but also comprehensively analyzed its mechanism from the perspective of nuclear translocation. Under normal circumstances, NF-κB forms a complex with its inhibitory protein IκBα, keeping NF-κB inactive in the cytoplasm. When cells are stimulated, such as by inflammatory mediators, IκBα protein is phosphorylated by specific kinases, and degraded, leading to the activation of NF-κB. Activated NF-κB enters the cell nucleus, binds to specific DNA sites, and transcriptionally regulates the expression of various inflammation-related genes, triggering an inflammatory response [26–28]. Fucoxanthin, as a natural compound, is believed to intervene in the IκBα/NF-κB signaling pathway. The research findings suggest that fucoxanthin inhibits the nuclear translocation of NF-κB [22,23], preventing NF-κB from entering the nucleus, thereby inhibiting the excessive release of inflammatory mediators and alleviating the severity of the inflammatory response. This finding reveals how fucoxanthin may exert its anti-inflammatory effects by regulating the IκBα/NF-κB signaling pathway.

Through an in-depth study of the mechanisms related to the IκBα/NF-κB signaling pathway, the researcher have gained a more comprehensive understanding of the role of fucoxanthin in inflammation regulation. This not only helps to reveal the anti-inflammatory mechanism of fucoxanthin but also provides new insights and evidence for the future design of more targeted anti-inflammatory drugs. However, the potential systemic toxicity and teratogenic risks associated with long-term high-dose fucoxanthin use remain poorly understood. Current beneficial effects of fucoxanthin are primarily limited to animal models, while long-term clinical data are still lacking. Species-specific differences and drug interactions require further clarification to ensure safe therapeutic applications. Future research directions could include further exploration of the dosage effects of fucoxanthin, its long-term safety, and its interactions with other signaling pathways.

## 5. Study strengths and limitations

This study is the first to discover that fucoxanthin has a significant anti-inflammatory effect on acne and has the value of becoming a novel therapeutic agent for acne. However, since the mechanisms of fucoxanthin are highly complex and involve multiple signaling pathways, this study is confined to its regulation of the IκBα/NF-κB signaling pathway. Whether it exerts protective effects through other pathways remains unelucidated. Future studies will further elucidate its mechanism of action, evaluate its clinical application value, and contribute to the development of more effective anti-inflammatory drugs.

## 6. Conclusions

Fucoxanthin exhibits significant anti-inflammatory effects by regulating the IκBα/NF-κB signaling pathway and inhibiting NF-κB nuclear translocation, offering a new research direction for the treatment of acne.

## Supporting information

S1 File S1_raw_images. The raw image of the blot.
(PDF)

S2 File S2_Supplementary_Figures. The supplementary figures of the article.
(DOCX)

S3 File S3_minimal data set. The minimal data set of the article.
(XLSX)

## References

[1] Dryden MS. Skin and soft tissue infection: microbiology and epidemiology. Int J Antimicrob Agents. 2009;34(Suppl 1):S2–7. doi: 10.1016/S0924-8579(09)70541-2 19560670

[2] Balighi K, Daneshpazhooh M, Lajevardi V, Talebi S, Azizpour A. Cheilitis in acne vulgaris patients with no previous use of systemic retinoid products. Australas J Dermatol. 2017;58(3):211–3. doi: 10.1111/ajd.12476 27003364

[3] Li X, Luo S, Chen X, Li S, Hao L, Yang D. Adipose-derived stem cells attenuate acne-related inflammation via suppression of NLRP3 inflammasome. Stem Cell Res Ther. 2022;13(1):334. doi: 10.1186/s13287-022-03007-7 35871079 PMC9308350

[4] Li F, Lin L, He Y, Sun G, Dong D, Wu B. BMAL1 regulates Propionibacterium acnes-induced skin inflammation via REV-ERBα in mice. Int J Biol Sci. 2022;18(6):2597–608. doi: 10.7150/ijbs.71719 35414779 PMC8990455

[5] Bharti S, Vadlamudi HC. A strategic review on the involvement of receptors, transcription factors and hormones in acne pathogenesis. J Recept Signal Transduct Res. 2021;41(2):105–16. doi: 10.1080/10799893.2020.1805626 32787477

[6] Tan AW, Tan H-H. Acne vulgaris: a review of antibiotic therapy. Expert Opin Pharmacother. 2005;6(3):409–18. doi: 10.1517/14656566.6.3.409 15794732

[7] Koshak MF, El-Readi MZ, Elzubier ME, Refaat B, Almaimani RA, Idris S, et al. Antioxidative and Anti-Inflammatory Protective Effects of Fucoxanthin against Paracetamol-Induced Hepatotoxicity in Rats. Mar Drugs. 2023;21(11):592. doi: 10.3390/md21110592 37999416 PMC10672227

[8] Chen Y, Dong J, Gong L, Hong Y, Hu C, Bao Y, et al. Fucoxanthin, a marine derived carotenoid, attenuates surgery-induced cognitive impairments via activating Akt and ERK pathways in aged mice. Phytomedicine. 2023;120:155043. doi: 10.1016/j.phymed.2023.155043 37639810

[9] Ben Ammar R, Zahra HA, Abu Zahra AM, Alfwuaires M, Abdulaziz Alamer S, Metwally AM, et al. Protective Effect of Fucoxanthin on Zearalenone-Induced Hepatic Damage through Nrf2 Mediated by PI3K/AKT Signaling. Mar Drugs. 2023;21(7):391. doi: 10.3390/md21070391 37504922 PMC10381773

[10] Chen Y, Lu H, Ding Y, Liu S, Ding Y, Lu B, et al. Dietary Protective Potential of Fucoxanthin as an Active Food Component on Neurological Disorders. J Agric Food Chem. 2023;71(8):3599–619. doi: 10.1021/acs.jafc.2c08249 36802555

[11] Martin LJ. Fucoxanthin and Its Metabolite Fucoxanthinol in Cancer Prevention and Treatment. Mar Drugs. 2015;13(8):4784–98. doi: 10.3390/md13084784 26264004 PMC4557004

[12] Wu S-J, Liou C-J, Chen Y-L, Cheng S-C, Huang W-C. Fucoxanthin Ameliorates Oxidative Stress and Airway Inflammation in Tracheal Epithelial Cells and Asthmatic Mice. Cells. 2021;10(6):1311. doi: 10.3390/cells10061311 34070405 PMC8227140

[13] Shih P-H, Shiue S-J, Chen C-N, Cheng S-W, Lin H-Y, Wu L-W, et al. Fucoidan and Fucoxanthin Attenuate Hepatic Steatosis and Inflammation of NAFLD through Modulation of Leptin/Adiponectin Axis. Mar Drugs. 2021;19(3):148. doi: 10.3390/md19030148 33809062 PMC8001566

[14] Su J, Guan B, Chen K, Feng Z, Guo K, Wang X, et al. Fucoxanthin Attenuates Inflammation via Interferon Regulatory Factor 3 (IRF3) to Improve Sepsis. J Agric Food Chem. 2023;71(33):12497–510. doi: 10.1021/acs.jafc.3c03247 37560933

[15] Urikura I, Sugawara T, Hirata T. Protective effect of Fucoxanthin against UVB-induced skin photoaging in hairless mice. Biosci Biotechnol Biochem. 2011;75(4):757–60. doi: 10.1271/bbb.110040 21512228

[16] Lee M-K, Ryu H, Lee JY, Jeong HH, Baek J, Van JY, et al. Potential Beneficial Effects of Sargassum spp. in Skin Aging. Mar Drugs. 2022;20(8):540. doi: 10.3390/md20080540 36005543 PMC9410049

[17] Liang W-J, Yang H-W, Liu H-N, Qian W, Chen X-L. HMGB1 upregulates NF-kB by inhibiting IKB-α and associates with diabetic retinopathy. Life Sci. 2020;241:117146. doi: 10.1016/j.lfs.2019.117146 31816325

[18] Wu Q, Zhou X, Li P, Ding M, You S, Xu Z, et al. ROC1 promotes the malignant progression of bladder cancer by regulating p-IκBα/NF-κB signaling. J Exp Clin Cancer Res. 2021;40(1):158. doi: 10.1186/s13046-021-01935-5 33962660 PMC8106150

[19] Kong L, Chen J, Ji X, Qin Q, Yang H, Liu D, et al. Alcoholic fatty liver disease inhibited the co-expression of Fmo5 and PPARα to activate the NF-κB signaling pathway, thereby reducing liver injury via inducing gut microbiota disturbance. J Exp Clin Cancer Res. 2021;40(1):18. doi: 10.1186/s13046-020-01782-w 33413501 PMC7788704

[20] Yu H, Lin L, Zhang Z, Zhang H, Hu H. Targeting NF-κB pathway for the therapy of diseases: mechanism and clinical study. Signal Transduct Target Ther. 2020;5(1):209. doi: 10.1038/s41392-020-00312-6 32958760 PMC7506548

[21] Lawrence T. The nuclear factor NF-kappaB pathway in inflammation. Cold Spring Harb Perspect Biol. 2009;1(6):a001651. doi: 10.1101/cshperspect.a001651 20457564 PMC2882124

[22] Lee C-Y, Chen S-P, Huang-Liu R, Gau S-Y, Li Y-C, Chen C-J, et al. Fucoxanthin decreases lipopolysaccharide-induced acute lung injury through the inhibition of RhoA activation and the NF-κB pathway. Environ Toxicol. 2022;37(9):2214–22. doi: 10.1002/tox.23587 35616142

[23] Yang Y-P, Tong Q-Y, Zheng S-H, Zhou M-D, Zeng Y-M, Zhou T-T. Anti-inflammatory effect of fucoxanthin on dextran sulfate sodium-induced colitis in mice. Nat Prod Res. 2020;34(12):1791–5. doi: 10.1080/14786419.2018.1528593 30488724

[24] Cui S, Wu H, He Q, Wang L, Yi X, Feng G, et al. Fucoxanthin alleviated atherosclerosis by regulating PI3K/AKT and TLR4/NFκB mediated pyroptosis in endothelial cells. Int Immunopharmacol. 2023;120:110370. doi: 10.1016/j.intimp.2023.110370 37235964

[25] Liu M, Li W, Chen Y, Wan X, Wang J. Fucoxanthin: A promising compound for human inflammation-related diseases. Life Sci. 2020;255:117850. doi: 10.1016/j.lfs.2020.117850 32470447

[26] Capece D, Verzella D, Flati I, Arboretto P, Cornice J, Franzoso G. NF-κB: blending metabolism, immunity, and inflammation. Trends Immunol. 2022;43(9):757–75. doi: 10.1016/j.it.2022.07.004 35965153

[27] Barnabei L, Laplantine E, Mbongo W, Rieux-Laucat F, Weil R. NF-κB: At the Borders of Autoimmunity and Inflammation. Front Immunol. 2021;12:716469. doi: 10.3389/fimmu.2021.716469 34434197 PMC8381650

[28] Zhang T, Ma C, Zhang Z, Zhang H, Hu H. NF-κB signaling in inflammation and cancer. MedComm (2020). 2021;2(4):618–53. doi: 10.1002/mco2.104 34977871 PMC8706767

