# Supplementary figures and images for: Fucoxanthin ameliorates Propionibacterium acnes-induced ear inflammation in mice by modulating the IκBα/NF-κB signaling pathway and inhibiting NF-κB nuclear translocation
Source: PLoS One. 2025 May 7;20(5):e0322950. doi: 10.1371/journal.pone.0322950 (PMC12057845; doi:10.1371/journal.pone.0322950)

## Raw Images

**Figure 2**

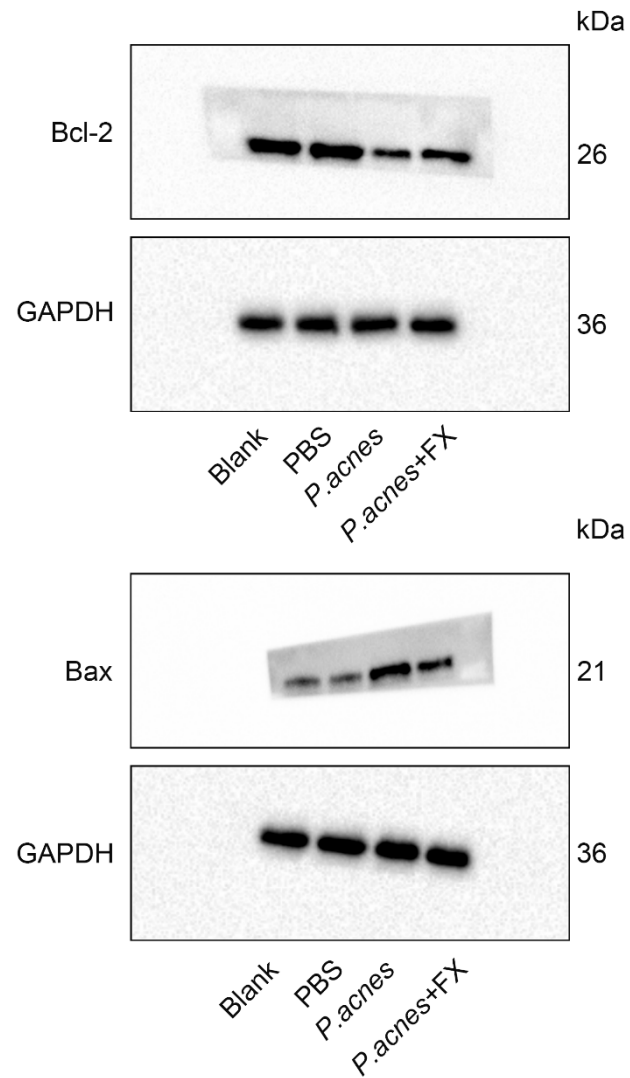

**Figure 3**

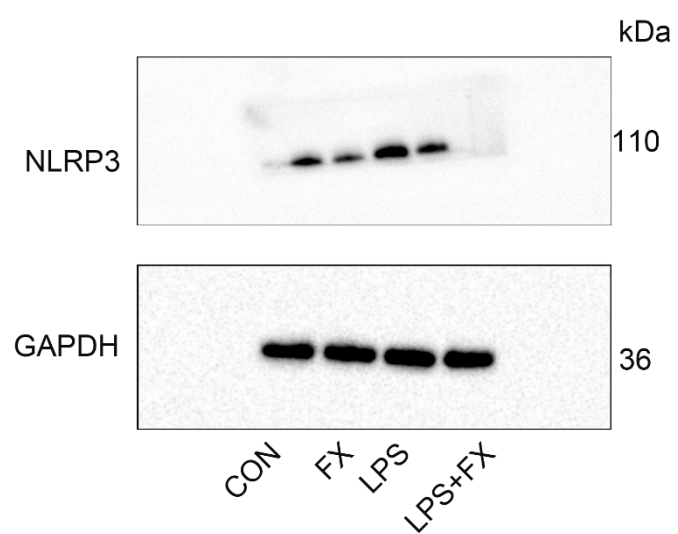

**Figure 4**

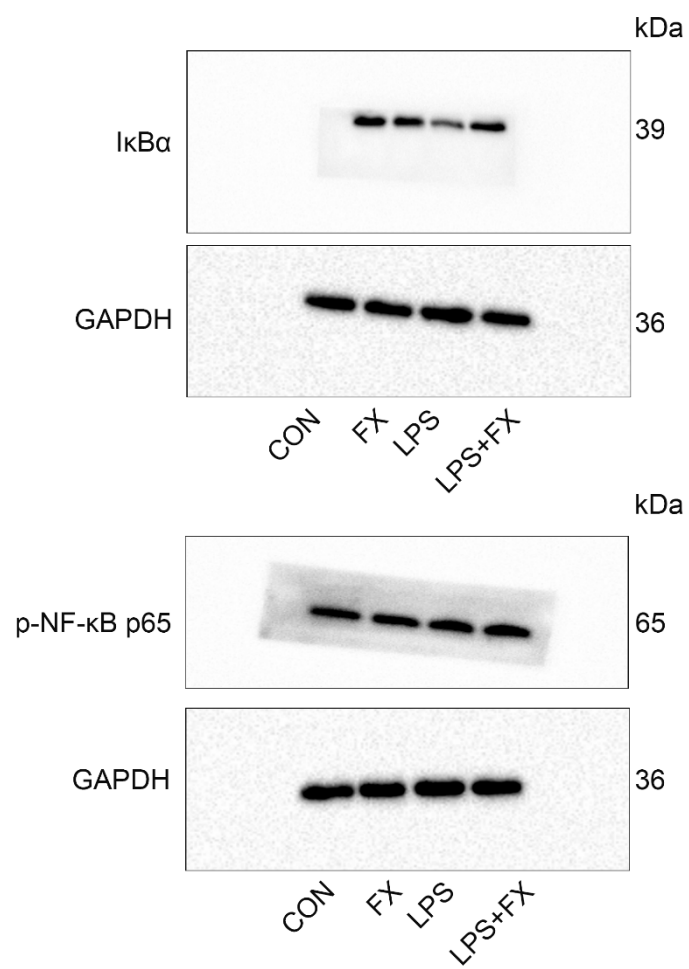

**Figure 5**

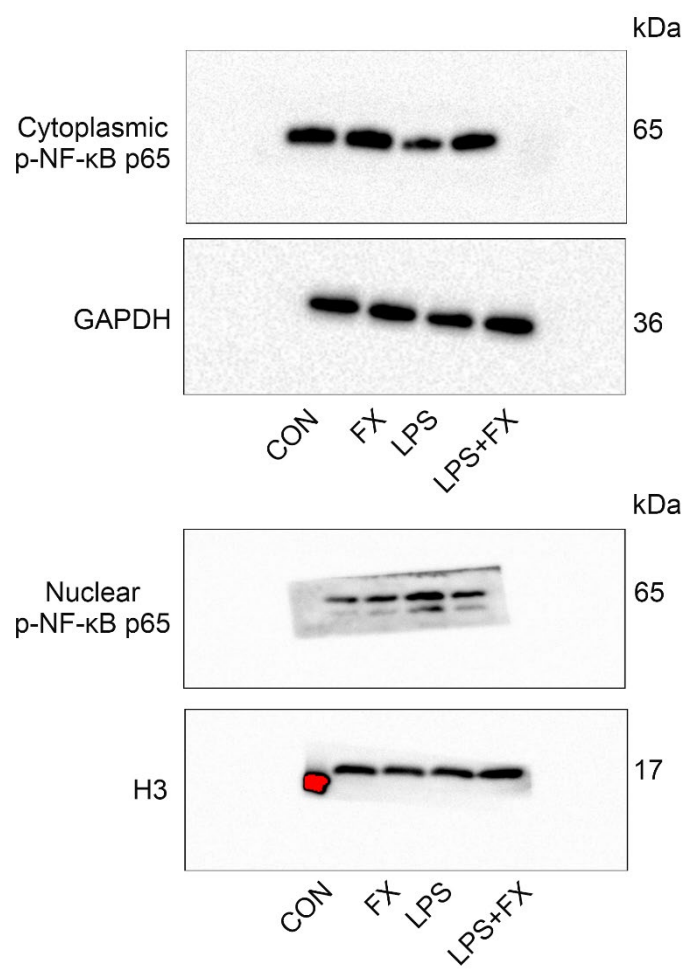

**Figure 6**

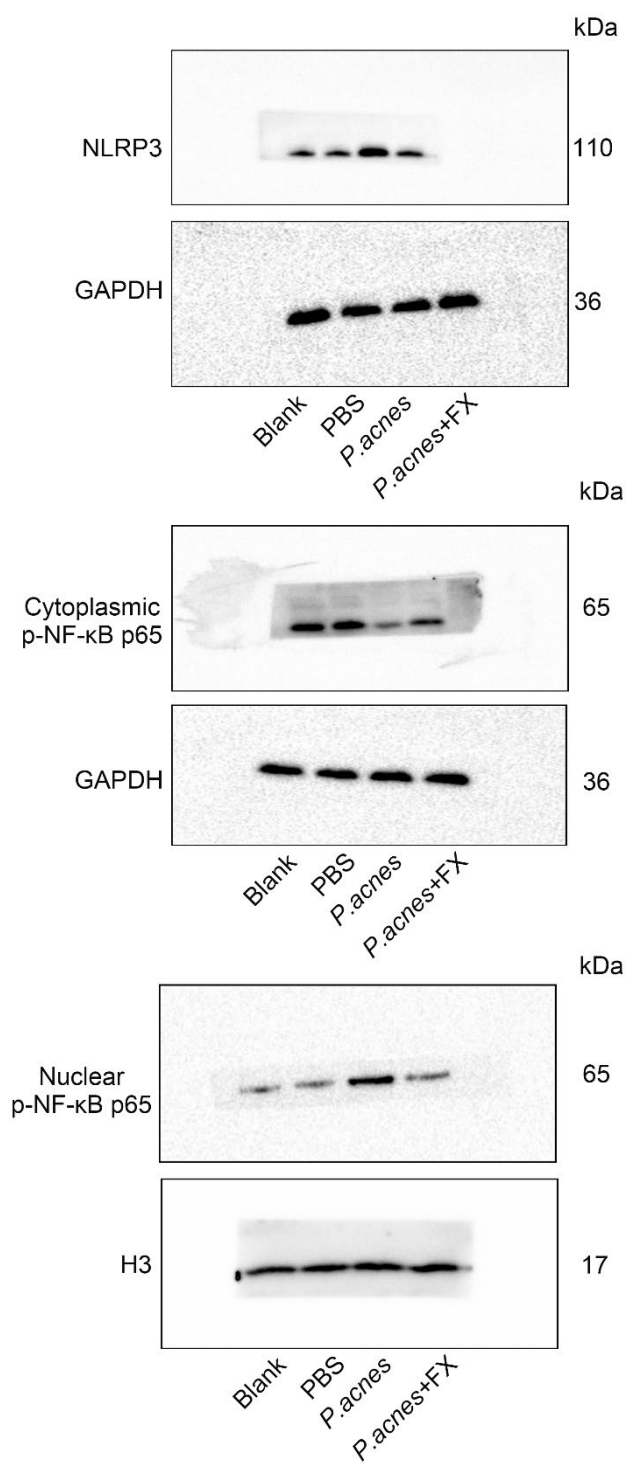

Supplement: S1 File — The raw image of the blot. (PDF) [file pone.0322950.s001.pdf]
