# Supplementary material for: Fucoxanthin ameliorates Propionibacterium acnes-induced ear inflammation in mice by modulating the IκBα/NF-κB signaling pathway and inhibiting NF-κB nuclear translocation
Source: PLoS One. 2025 May 7;20(5):e0322950. doi: 10.1371/journal.pone.0322950 (PMC12057845; doi:10.1371/journal.pone.0322950)
Supplement: S2 File — The supplementary figures of the article. (DOCX) [file pone.0322950.s002.docx]

**Supplementary Figures**

**Supplementary Figure 1**


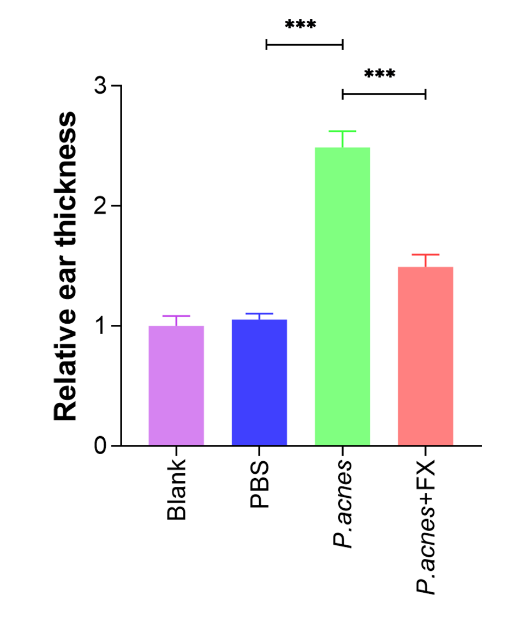


Supplementary Figure 1: The relative thickness of the ear in mice. Data are expressed as mean ± SD, n=6. ^***^ *P* <0.001.

**Supplementary Figure 2**


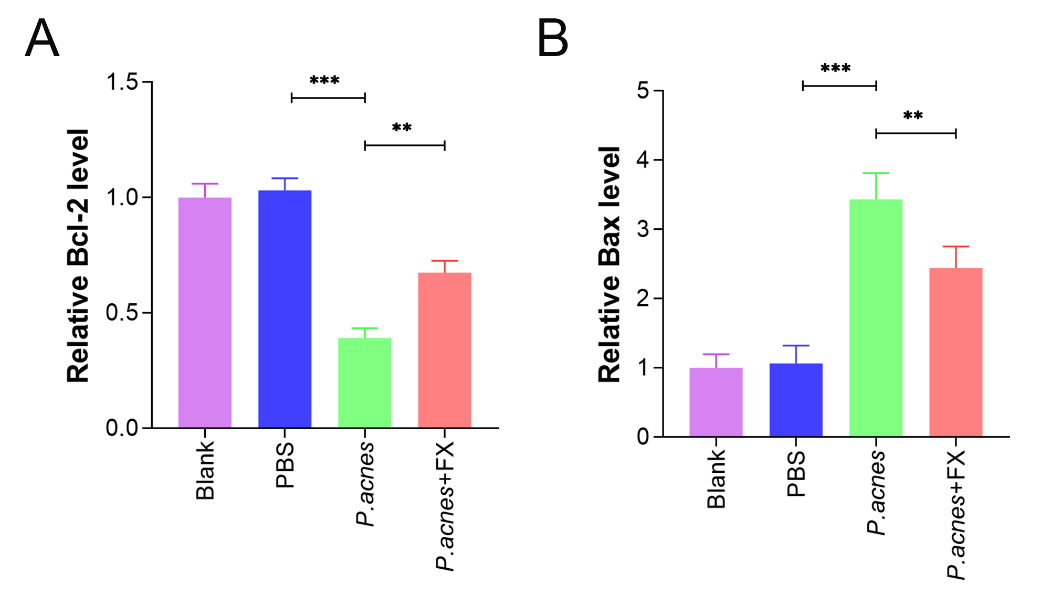


Supplementary Figure 2: The relative expression levels of Bcl-2 and Bax protein in the ear of mice.

A, The relative expression levels of Bcl-2 protein; B, The relative expression levels of Bax protein. Data are expressed as mean ± SD, n=3. ^***^ *P* <0.001, ^**^ *P* <0.01.

**Supplementary Figure 3**


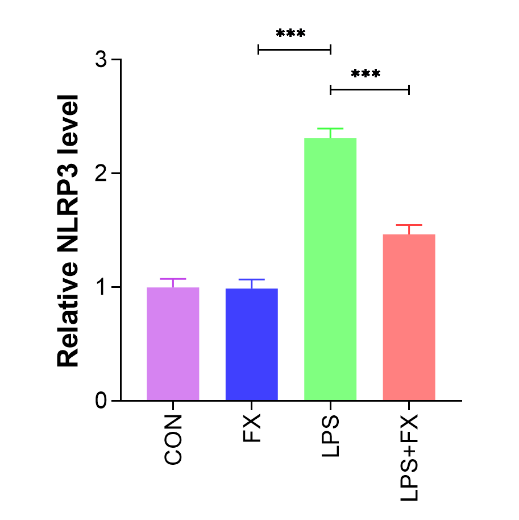


Supplementary Figure 3: The relative expression levels of NLRP3 protein in HaCaT cells.

Data are expressed as mean ± SD, n=3. ^***^ *P* <0.001.

**Supplementary Figure 4**


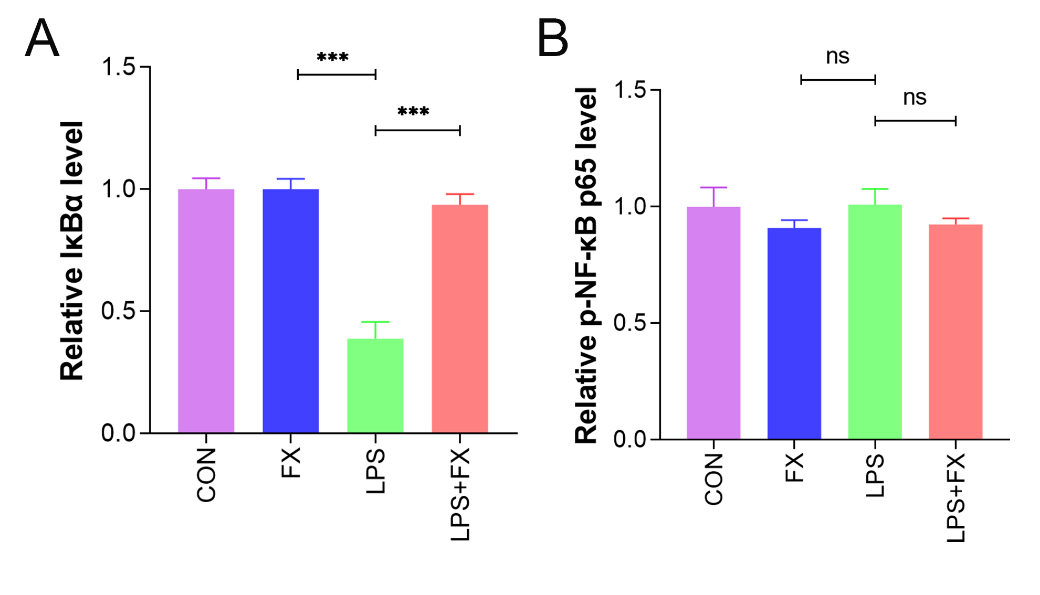


Supplementary Figure 4: The relative expression levels of IκBα and NF-κB protein in HaCaT cells.

A, The relative expression levels of IκBα protein; B, The relative expression levels of p-NF-κB p65 protein. Data are expressed as mean ± SD, n=3. ^***^ *P* <0.001, ^ns^ *P*＞0.05.

**Supplementary Figure 5**


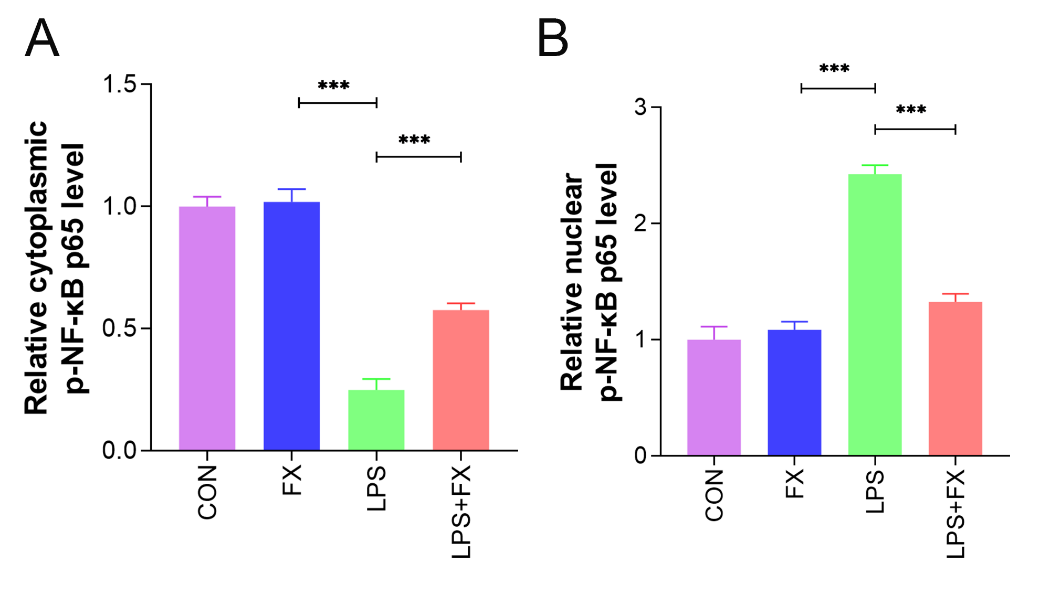


Supplementary Figure 5: The relative expression levels of NF-κB protein in HaCaT cells.

A, The relative expression levels of cytoplasmic p-NF-κB p65 protein; B, The relative expression levels of nuclear p-NF-κB p65 protein. Data are expressed as mean ± SD, n=3. ^***^ *P* <0.001.

**Supplementary Figure 6**


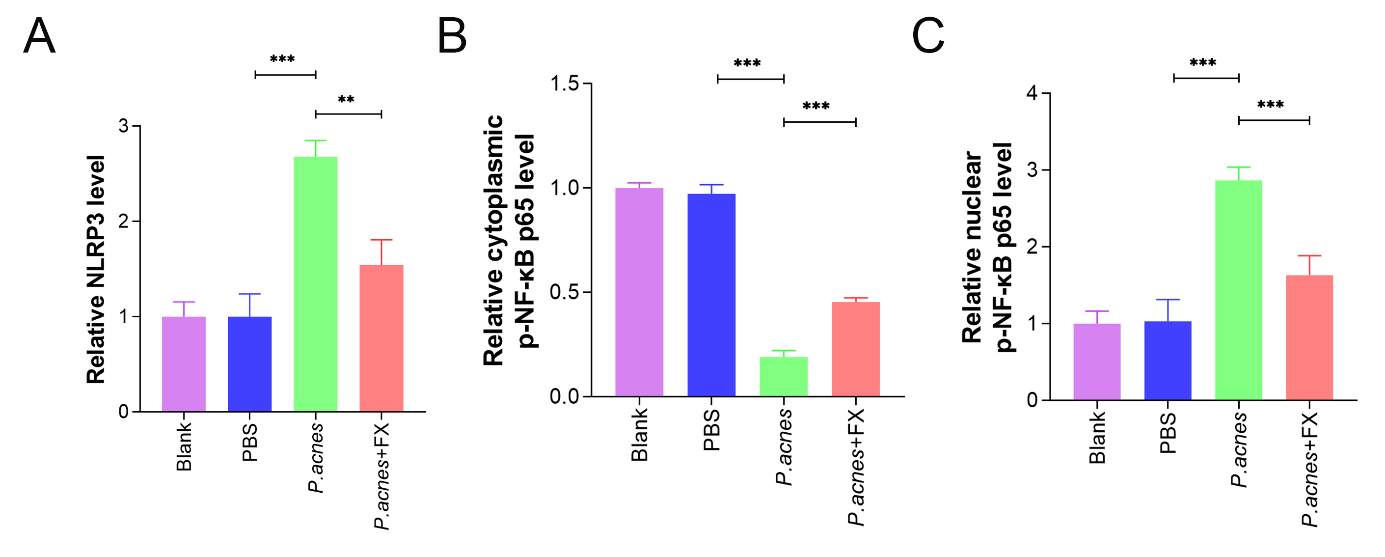


Supplementary Figure 6: The relative expression levels of NLRP3 and NF-κB protein in the ear of mice.

A, The relative expression levels of NLRP3 protein; B, The relative expression levels of cytoplasmic p-NF-κB p65 protein; C, The relative expression levels of nuclear p-NF-κB p65 protein. Data are expressed as mean ± SD, n=3. ^***^ *P* <0.001.
